# WHI-2 Regulates Intercellular Communication via a MAP Kinase Signaling Complex

**DOI:** 10.3389/fmicb.2019.03162

**Published:** 2020-01-22

**Authors:** A. Pedro Gonçalves, Karen M. Chow, Sara Cea-Sánchez, N. Louise Glass

**Affiliations:** ^1^Department of Plant and Microbial Biology, University of California, Berkeley, Berkeley, CA, United States; ^2^Departamento de Genética, Universidad de Sevilla, Sevilla, Spain; ^3^Environmental Genomics and Systems Biology Division, Lawrence Berkeley National Laboratory, Berkeley, CA, United States

**Keywords:** cell fusion, WHI-2, CSP-6, AMPH-1, endocytosis, MAPK

## Abstract

The formation of the fungal mycelial network is facilitated by somatic cell fusion of germinating asexual spores (or germlings). *Neurospora crassa* germlings in close proximity display chemotropic growth that is dependent upon an intracellular network of mitogen-activated protein kinase (MAPK) signaling cascades. Approximately 80 genes involved in intercellular communication and fusion have been identified, including three mutants with similar morphological phenotypes: Δ*whi-2*, Δ*csp-6*, and Δ*amph-1*. Here we show that WHI-2 localizes to the cell periphery and regulates endocytosis, mitochondrial organization, sporulation, and cell fusion. WHI-2 was required to transduce signals through a conserved MAPK pathway (NRC-1/MEK-2/MAK-2) and target transcription factors (PP-1/ADV-1). The *amph-1* locus encodes a Bin/Amphiphysin/Rvs domain-containing protein and mis-expression of *whi-2* compensated for the cell fusion and endocytosis deficiencies of a Δ*amph-1* mutant. The *csp-6* locus encodes a haloacid dehalogenase phosphatase whose activity was essential for cell fusion. Although fusion-deficient with themselves, cells that lacked *whi-2, csp-6*, or *amph-1* showed a low frequency of chemotropic interactions with wild type cells. We hypothesize that WHI-2 could be important for signal perception during chemotropic interactions via a role in endocytosis.

## Introduction

Fungi can sense their surroundings, receive environmental cues, interpret them, and respond accordingly. During asexual growth, germinated asexual spores (germlings) and hyphae of the ascomycete fungus *Neurospora crassa* display social behaviors that trigger the process of cell-cell communication and somatic cell fusion. Cell fusion creates a mycelial network that allows the circulation of nutrients, water and cellular elements including genetic material throughout an interconnected colony (Leeder et al., 2011; Fischer and Glass, 2019). Somatic cell fusion in *N. crassa* operates in an analogous way to somatic cell fusion events in mammalian systems, including during muscle, placenta, and bone tissue development (Hernández and Podbilewicz, 2017).

In *N. crassa*, somatic cell fusion of germlings is initiated when two cells (which can be genetically identical) undergo chemotropic interactions via the exchange of yet-to-be discovered signals by forming polarized cellular protrusions called conidial anastomosis tubes (CATs) (Gabriela Roca et al., 2005). After CATs from germlings come into contact, a switch from cell growth to cell wall dissolution is initiated (Gonçalves et al., 2019), followed by plasma membrane merger and cytoplasmic continuity; nuclear fusion is not a consequence of somatic cell fusion (Leeder et al., 2011). The combination of polar growth, hyphal branching, and cell fusion events is vital for the formation of the interconnected multicellular hyphal network that is the hallmark growth habit of filamentous fungi (Glass et al., 2004).

The availability of a well-annotated genome (Galagan et al., 2003; Borkovich et al., 2004) and a near full genome deletion strain collection (Colot et al., 2006) have enabled screening of deletion mutants for cell fusion phenotypes in *N. crassa* (Fu et al., 2011). Deletion strains affected in cell communication and fusion often show a reduction in aerial hyphae extension and flat-like growth and approximately 80 genes have been found to play a role in these processes in *N. crassa*. The functions of these genes have been partially characterized and range from intracellular signaling, calcium modulation, membrane merger, production of reactive oxygen species, actin regulation, vesicle trafficking and transcriptional control (Glass et al., 2004; Leeder et al., 2011; Fischer and Glass, 2019). Two conserved mitogen-activated protein kinase (MAPK) modules form the core of the intracellular signal transduction network that is activated during cell fusion: the SOFT (SO)/MIK-1/MEK-1/MAK-1 cascade—that is part of the cell wall integrity pathway—and the STE-50/STE-20/HAM-5/NRC-1/MEK-2/MAK-2 module (Fischer and Glass, 2019). During chemotropic growth that precedes cell fusion, components of both of these pathways are recruited to the tip of germling CATs and to the tips of fusion hyphae (Fleissner et al., 2009b; Dettmann et al., 2012, 2014; Jonkers et al., 2014). The tip recruitment and displacement of the MAK-2 signaling module to a single fusion tip occurs every ~8–10 min and alternates at CAT and fusion tips of chemotropic partners in a perfectly out of phase manner with localization of SO, resembling a ping-pong mechanism of signal sending and receiving (Fleissner et al., 2009b; Leeder et al., 2011; Serrano et al., 2018). These two pathways are required for the activation of the transcription factors PP-1 and ADV-1 that function as master regulators of cell fusion genes (Fischer et al., 2018).

Despite recent advances, the function of many cell fusion genes remains unclear. In particular, *whi-2*, the *N. crassa* ortholog of the *Saccharomyces cerevisiae* gene *WHISKEY2* (*WHI2*), has been shown to be required for cell fusion (Fu et al., 2014). The *S. cerevisiae* Whi2 was initially identified as a central player in the coordination between cell proliferation and nutrient availability. Loss-of-function mutations in *WHI2* result in cells that cannot properly sense the extracellular nutritional status and fail to shift from exponential to stationary growth (Saul and Sudbery, 1985), leading to cells that are smaller than normal due to cell division without sustained cell growth. Yeast *whi2* mutants also show actin cytoskeleton disorganization, increased cell death, aberrant mitochondrial morphology and defects in executing endocytosis (Binley et al., 1999; Care et al., 2004; Leadsham et al., 2009). Additionally, *whi2* mutants show an increase in the activity of the Ras/cAMP/PKA pathway, permitting the downstream general stress response transcription factor Msn2/4 to remain phosphorylated and outside of the nucleus, where it is unable to activate the expression of cell cycle arrest genes (Radcliffe et al., 1997a; Leadsham et al., 2009; Sadeh et al., 2011). Overexpression of *WHI2* results in filamentous growth that is dependent on Ste11 and partially dependent on Ste7, Ste20 and Ste12 (Radcliffe et al., 1997b). These proteins are orthologs of *N. crassa* NRC-1 (Ste11), MEK-2 (Ste7), STE-20 (Ste20), and PP-1 (Ste12), components of a MAPK signaling complex and its target transcription factor that are required for somatic cell fusion in a number of filamentous fungi (Fischer and Glass, 2019). The *S. cerevisiae WHI2* gene also seems to be a hot spot for adaptive mutations, indicating a central role for the respective protein in fungal development and environmental responses (Cheng et al., 2008; Gresham et al., 2008; Kvitek and Sherlock, 2013; Lang et al., 2013; Teng et al., 2013; Szamecz et al., 2014; Treusch et al., 2015; Payen et al., 2016; Comyn et al., 2017).

In this study, we examined the role of the *N. crassa* WHI-2 during fungal development. Our findings indicate that WHI-2 localized to the cell periphery, affected endocytosis and mitochondrial morphology, and functioned upstream of the NRC-1/MEK-2/MAK-2/PP-1/ADV-1 signaling pathway to activate cell-cell communication. Additionally, we discuss two other cell fusion proteins, CSP-6 and AMPH-1, whose functions are related to WHI-2. CSP-6 showed a localization pattern similar to WHI-2 and its deletion phenocopied the morphological and cell fusion defects of a Δ*whi-2* strain. AMPH-1 is an endocytosis regulator whose absence was compensated for by the mis-expression of WHI-2. This work provides new details on genes/proteins that function upstream of a key MAPK signaling module.

## Materials and Methods

### Strains and Culture Media

Standard procedures for the handling of *N. crassa* cells were employed. Cells were grown in Vogel's minimal medium (VMM) plus 2% (w/v) sucrose and 1.5% (w/v) agar (Vogel, 1956). Crosses were performed on synthetic cross medium (Westergaard and Mitchell, 1947). Wild type and deletion strains are available from the Fungal Genetics Stock Center (FGSC) (McCluskey et al., 2010) and were constructed as part of the Neurospora Genome Project (Colot et al., 2006). Strains used in this study are listed in Table S1. For all the experiments, the indicated strains were grown for ~7 days in VMM-containing slant tubes and a conidial suspension was obtained by adding sterile _dd_H_2_O into tubes, vortexing and passing the mixture through cheesecloth to remove hyphal fragments. Hygromycin B (Thermo Fisher Scientific, Waltham, MA, USA) was used at a final concentration of 200 μg/ml.

### Strain Construction

The *whi-2, csp-6*, and *amph-1* genes were amplified from genomic DNA of the FGSC2489 wild type strain using primers ACCTCTAGAATGGCTGCCGCGGGAGGAG and CAGTTAATTAAACGCAGTCCAATCACACTCATCTCC for *whi-2* (creating XbaI/PacI restriction sites), primers TTTTACTAGTATGAGCAACTCGAACCCG and TTTTTTAATTAAAAGAGTGACGTCCAGAACCAG for *csp-6* (creating SpeI/PacI restriction sites) and primers TTTTTTAATTAAAAGAGTGACGTCCAGAACCAG and CGGTTAATTAAAACAGTTCCGCTGATACTC for *amph-1* (creating XbaI/PacI restriction sites). The resulting PCR products were cloned into pCR-Blunt II-TOPO, excised using the respective combination of restriction enzymes and inserted into pMF272 (GenBank accession number: AY598428.1) (Freitag et al., 2004). In pMF272, *whi-2, csp-6* and *amph-1* were placed downstream of a *ccg-1* promoter and upstream of *sgfp* and a *ccg-1* terminator. For construction of the CSP-6^D284A^ strain, site-directed mutagenesis using primers ccttgttctaGCTttggatgaaa and tttcatccaaAGCtagaacaagg were used. These primers were used in a Pfu-based PCR using the pMF272 vector carrying the wild type *csp-6* (described above) as the DNA template that was followed by treatment with the DpnI restriction enzyme. The resulting vectors were transformed into Δ*whi-2*; *his-3*, Δ*csp-6*; *his-3* and Δ*amph-1*; *his-3* conidia, respectively, using a Gene Pulser electroporator (Bio-Rad, Hercules, CA, USA) at 1.5 kV, 25 μF, 600 Ω. Homokaryotic strains expressing the indicated *gfp*-tagged genes were obtained by backcrosses. Sanger sequencing to confirm that there were no irregularities in the constructs was performed at the UC Berkeley DNA Sequencing Facility.

### Microscopy

Conidia were diluted to a concentration of 1.5 × 10^7^ cells/ml. For the evaluation of conidial morphology, 10 μl were pipetted onto a glass slide and covered with a coverslip. To examine communication frequency, 80 μl of conidial suspension was spread onto 5 cm VMM agar plates. In co-culture experiments two strains were mixed in a 1:1 proportion before plating. Staining with FM4-64 (*N*-(3-triethylammoniumpropyl)-4-(4-diethylaminophenylhexatrienyl) pyridinium dibromide; Thermo Fisher Scientific, Waltham, MA, USA) was carried out by incubating 2 μM FM4-64 in a 1.5 × 10^7^ cells/ml conidial suspension in a total volume of 500 μl for 15 min in the dark; the cells were then washed twice with _dd_H_2_O and resuspended in 500 μl to reestablish the initial spore concentration. The plates were briefly dried in a fume hood and incubated at 30°C, in the dark for 3.5–4 h or 16–20 h to analyze cells at the germling or hyphal stage, respectively. Squares of ~1 cm were excised and observed. For the analysis of the accumulation of endocytic intermediates, 15 μl of 4 μM FM4-64 was added to the agar slice immediately before imaging. Conidial morphology and cell communication were assessed using a Zeiss Axioskop 2 using a 40x Plan-Neofluor oil immersion objective lens. The percentage of cell communication was determined by counting the relative frequency of cell pairs that displayed a chemotropic behavior when germinated conidia were within ~15 μm of each other. For the FM4-64 microscopy assay, as well as WHI-2, CSP-6, AMPH-1, ARG-4, MAK-2 and SO localization studies, a Leica SD6000 confocal microscope equipped with a Yokogawa CSU-X1 spinning disk head, 488 nm and 561 nm lasers and a 100 × 1.4 N.A. oil-immersion objective lens controlled by Metamorph (Molecular Devices, LLC, San Jose, CA, USA) was used. Images were analyzed using ImageJ (Schneider et al., 2012). Multiple cells were analyzed per experiment and representative examples are shown.

### Flow Cytometry

In order to examine if plasma membrane material was being appropriately guided to the vacuoles by endocytosis, we adapted a previously published method (Zheng et al., 1998). Conidia at a concentration of 10^6^/ml were inoculated into glass tubes containing 1.5 ml VMM without agar and grown at 30°C, 200 rpm, for a total duration of 4 hrs; 2 μM FM4-64 and 10 μM 7-amino-4-chloromethylcoumarin (CMAC Blue; Thermo Fisher Scientific, Waltham, MA, USA) were added to the cultures for the last 45 and 15 min, respectively. The conidia were harvested by centrifugation (5,000 rpm, 5 min, 4°C) and washed twice with cold 1x PBS before being resuspended in cold 1x PBS and at least 10,000 events acquired on a BD LSRFortessa X-20 flow cytometer (BD Biosciences, Franklin Lakes, NJ, USA). The fluorescence of FM4-64 was recorded using a 488 nm laser and 685LP (710/50 nm) filter; the fluorescence of CMAC Blue was recorded using a 355 nm laser and (515/30 nm) filter. FlowJo (FlowJo, LLC, Ashland, OR, USA) was used for analyses.

### Bioinformatics and Statistical Analysis

The presence of conserved domains was assessed using InterProScan 5 (Jones et al., 2014). The WHI-2/Whi2p amino acid alignment was edited using BoxShade (https://embnet.vital-it.ch/software/BOX_form.html). The percentage of identity and similarity was calculated using the Sequence Manipulation Suite (http://www.bioinformatics.org/sms2/ident_sim.html). Statistical significance was tested by ANOVA followed by a Tukey *post-hoc* test using Prism (GraphPad Software, San Diego, CA, USA). At least three independent experiments were performed for all data shown in this paper. The schematic model of the function of WHI-2, CSP-6, and AMPH-1 was built using BioRender (app.biorender.com).

## Results

### WHI-2 Is Epistatic to the NRC-1/MEK-2/MAK-2 Signaling Pathway

**Figure 1:**
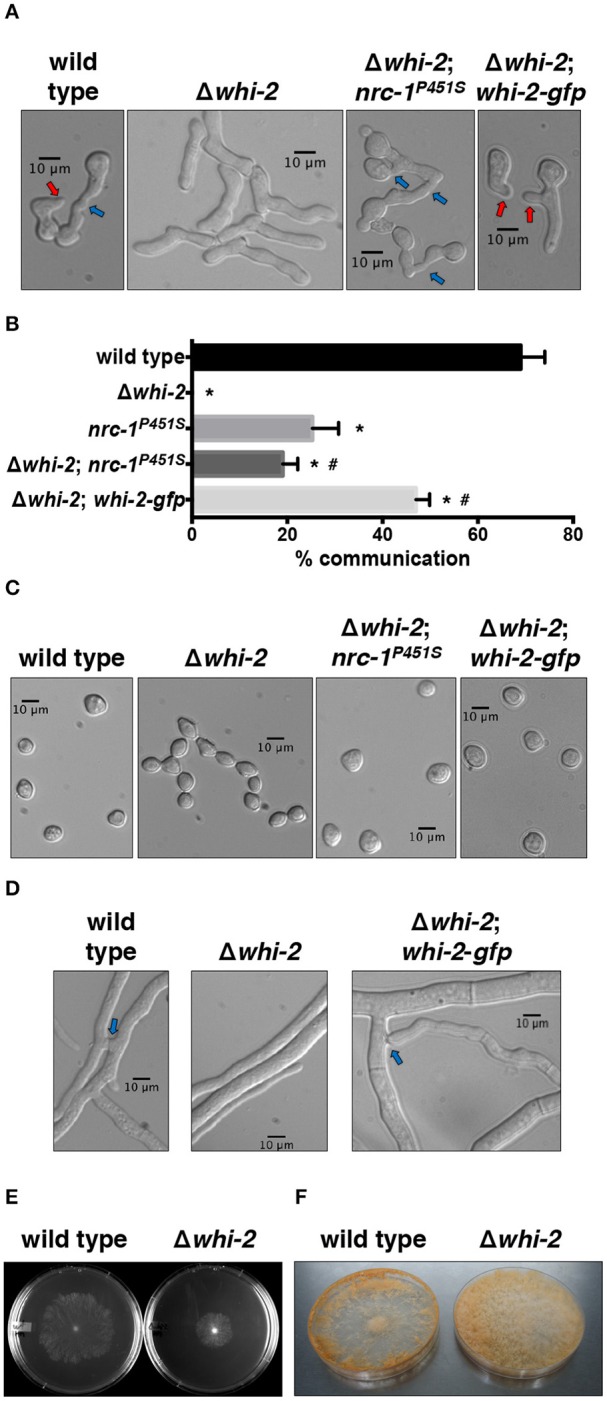
Cell fusion and conidiogenesis defects observed in a Δ*whi-2* mutant can be partially rescued by the introduction of a constitutively active allele of *nrc-1*. **(A,B)** Cell-cell communication was analyzed by observing brightfield microscopy **(A)** of closely positioned germlings 4 h post-inoculation. Red and blue arrows indicate conidial anastomosis tubes (CATs) and cell communication/fusion events, respectively. The relative frequency of cell-cell communication is shown in **(B)**. Error bars represent the standard deviation. *p-value < 0.0001 vs. wild type; #*p*-value < 0.001 vs. Δ*whi-2*. **(C)** Conidial morphology was assessed in WT and *whi-2* strains using brightfield microscopy. Note the presence of proconidial chains in the Δ*whi-2* strain. **(D)** Hyphal fusion was evaluated 16 h post-inoculation. Blue arrows point to hyphal fusion events. **(E,F)** Radial growth **(E)** and sporulation **(F)** were analyzed in wild type and Δ*whi-2* colonies 24 h or 7 days after inoculation on Petri dishes, respectively.

The *N. crassa* WHI-2 (NCU10518) is a 297 amino acid protein that harbors two BTB (Broad-complex, Tramtrack, and Bric-à-brac)/POZ (POx virus and Zinc finger) conserved domains (Figure S1). These domains have been shown to be involved in protein-protein interactions during multiple cellular processes ranging from ion channel assembly and gating, actin dynamics, transcriptional regulation to chromatin remodeling (Perez-Torrado et al., 2006). A recent report suggested that *whi-2* homologs might be distant relatives of members of the human disease-related potassium channel tetramerization domain (KCTD) protein family (Teng et al., 2018). *N. crassa* WHI-2 shows 24% identity and 33.5% similarity to *S. cerevisiae* Whi2p, but is substantially shorter (297 aa compared to 486 aa) (Figure S1). Previous deletion strain screenings in *N. crassa* identified multiple genes, including *whi-2*, as being required for germling fusion (Chinnici et al., 2014; Fu et al., 2014). In addition to the germling fusion defect (Figures 1A,B), the Δ*whi-2* deletion strain displayed additional morphological defects (Fu et al., 2014), such as an impairment in the formation of aerial hyphae (Figure S2), production of unseparated proconidial chains (Figure 1C) and lack of hyphal fusion (Figure 1D). The introduction of an epitope-tagged *whi-2* allele (*whi-2-gfp)* in the Δ*whi-2* mutant complemented the germling and hyphal fusion (Figures 1A,B,D), conidial separation (Figure 1C) and aerial hyphae (Figure S2) developmental defects. The Δ*whi-2* mutant grew slower than the wild type strain (Figure 1E) and produced profuse spores across a Petri plate, as compared to the wild type strain that sporulated mainly around the perimeter of the plate (Figure 1F). The Δ*whi-2* strain also did not produce protoperithecia when grown on synthetic cross medium, as shown in a previous deletion collection screening (Fu et al., 2014). Fertility was restored in Δ*whi-2*; *whi-2-gfp* cells and this strain was used as a female in crosses in subsequent experiments (Table S1).

**Figure 2:**
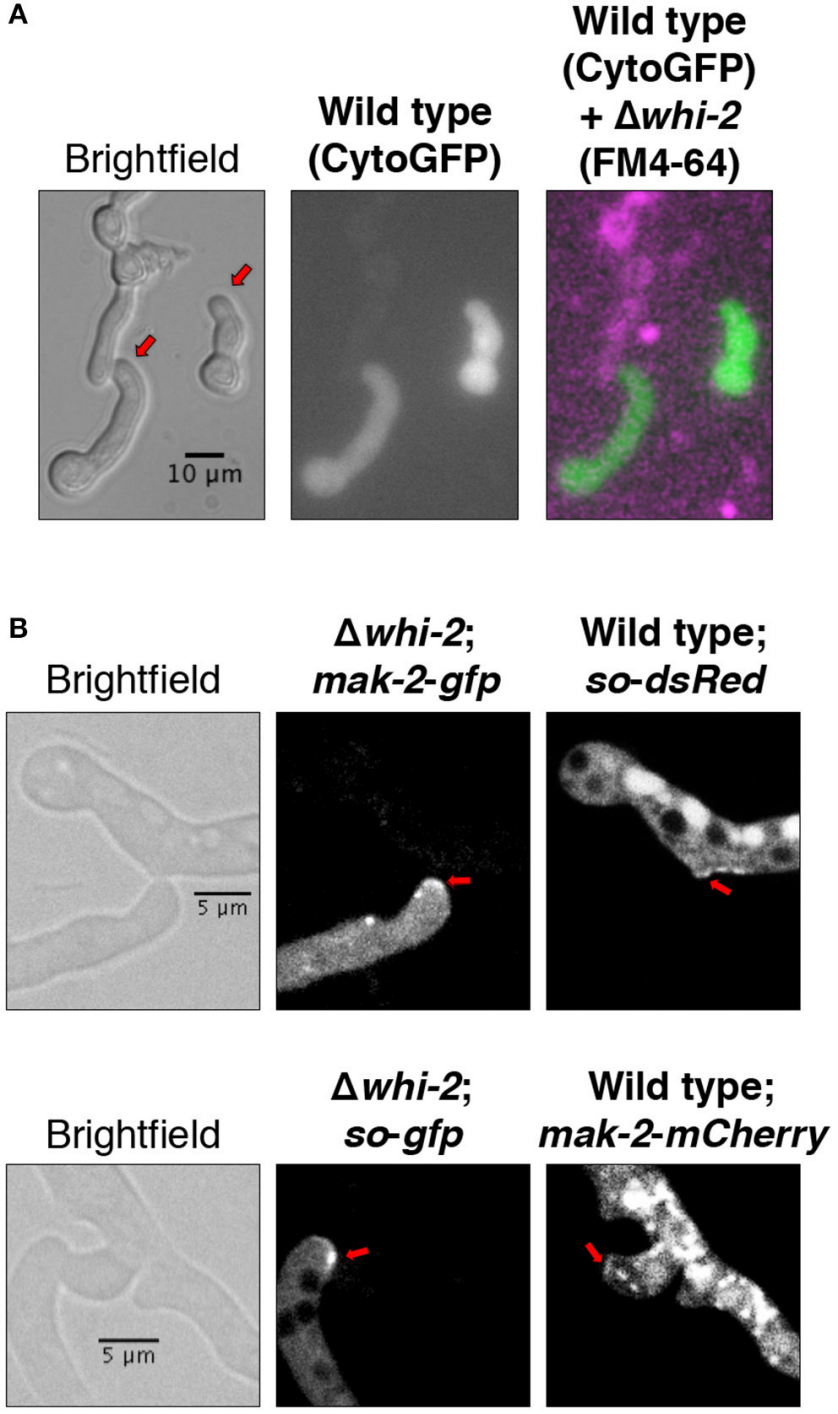
Cells lacking *whi-2* are capable of establishing chemotropic interactions with the wild type cells and show recruitment of MAK-2 and SO to CATs. **(A)** Cytoplasmic GFP-expressing wild type cells were mixed with FM4-64-stained Δ*whi-2* cells and observed by microscopy after germination. **(B)** MAK-2-GFP-expressing (upper panel) or SO-GFP-expressing (lower panel) Δ*whi-2* cells were mixed, respectively, with SO-dsRed-expressing or MAK-2-mCherry-expressing wild type cells and observed by microscopy after germination. Note the formation of CATs (red arrows).

Existing data suggests that Δ*whi-2* cells can communicate with wild type partner cells at a low frequency (Fu et al., 2014), a phenotype that we confirmed in Δ*whi-2*/wild type germling pairs (Figure 2A). When MAK-2-GFP or SO-GFP were expressed in the Δ*whi-2* mutant, recruitment of both proteins to the CATs of Δ*whi-2* cells undergoing chemotropic interactions with the wild type cells was observed (Figure 2B). Thus, the absence of WHI-2 did not directly affect the ability to form CATs nor the recruitment of MAK-2 and SO to communicating cell tips in wild type + Δ*whi-2* pairings.

Phosphorylation of the MAP kinases MAK-1 and MAK-2 is reduced in Δ*whi-2* germlings (Fu et al., 2014). To further explore the relationship between the MAK-2 pathway and WHI-2, we utilized a gain-of-function mutation *nrc-1* allele (proline to serine mutation at position 451 of NRC-1). Strains containing this allele show a ~12-fold increase in MAK-2 phosphorylation (Dettmann et al., 2012). When the *nrc-1*^*P*451*S*^ allele was introduced into the Δ*whi-2* mutant, the formation of proconidial chains was suppressed and cell fusion-associated chemotropism was restored to levels similar to an *nrc-1*^*P*451*S*^ strain (Figures 1A–C). At the macroscopic level, aerial hyphae development was also partially restored in the Δ*whi-2* mutant expressing *nrc-1*^*P*451*S*^ (Figure S2). These data place WHI-2 upstream of the STE-50/STE-20/HAM-5/NRC-1/MEK-2/MAK-2/PP-1/ADV-1 signal transduction pathway.

### Deletion of *whi-2* Results in Cells That Accumulate Endocytic Intermediates and Possess a Defective Mitochondrial Network

**Figure 3:**
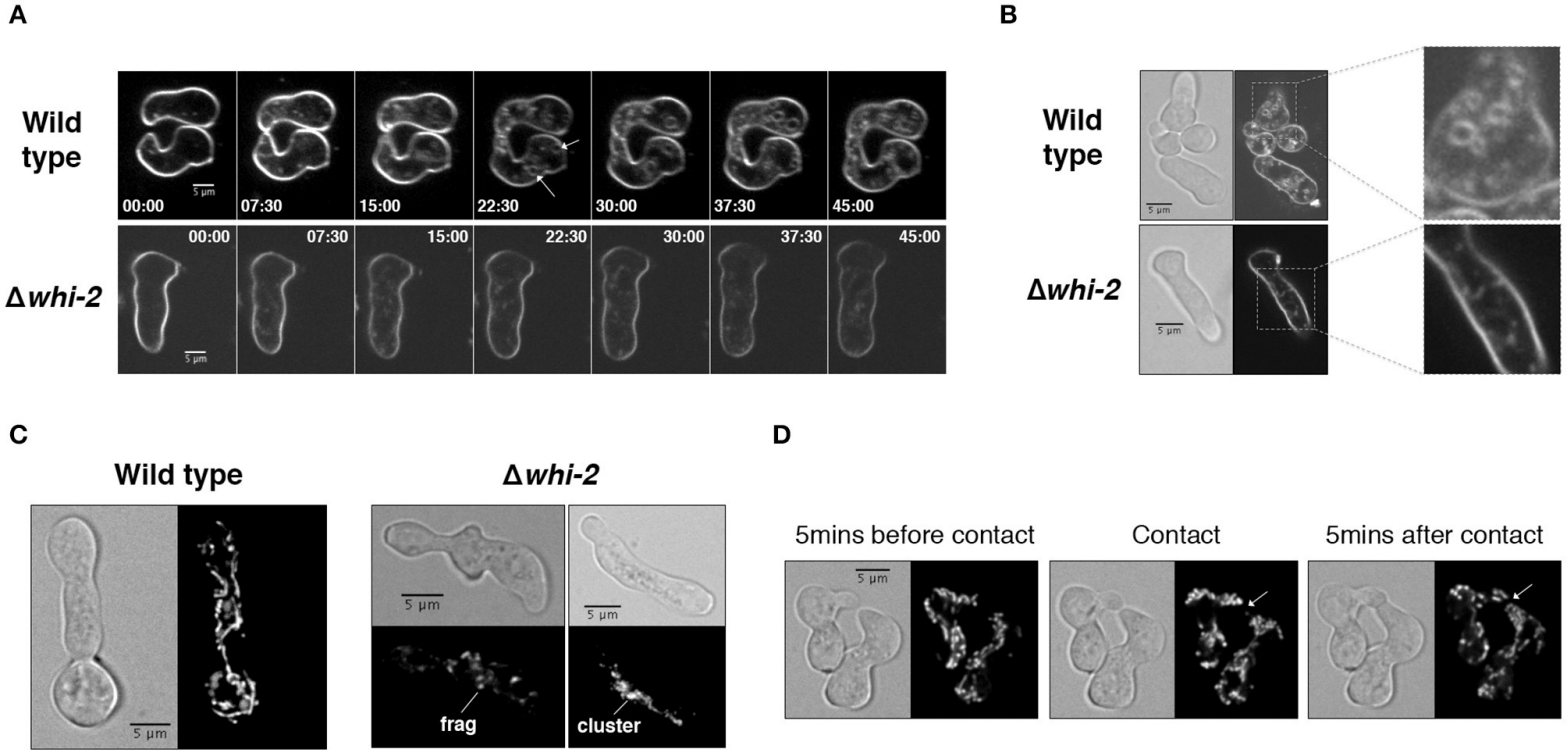
Strains lacking *whi-2* are defective in the accumulation of FM4-64-positive membranous structures and show defects in mitochondrial dynamics. **(A,B)** The uptake of FM4-64 via plasma membrane-derived structures was followed over time in wild type and Δ*whi-2* germlings. A time course experiment is shown in **(A)**. Note the formation of large FM4-64-positive membranous structures in the wild type strain (arrows). Representative images after 30 min of incubation are shown in **(B)**. These panels are complemented by Video 1. **(C)** The organization of the mitochondrial network was examined using an ARG-4-GFP construct (Bowman et al., 2009). Note the appearance of fragmented (“frag”) and clusters (“cluster”) of mitochondria in Δ*whi-2* cells. **(D)** Mitochondrial dynamics during cell fusion were evaluated by producing time-lapse videos of interacting wild type germlings. Note the absence of mitochondria at the fusion spot during the first minutes after contact (arrow). This panel is complemented by Video 2.

In *S. cerevisiae*, the deletion of *WHI2* causes a defect in endocytosis (Care et al., 2004). Here we employed the lipophilic styryl dye FM4-64 to track the accumulation of endocytic intermediates in *N. crassa* germlings. FM4-64 is unable to freely cross membranes; instead it is anchored on the outer leaflet of the plasma membrane bilayer. When endocytic membranous structures are formed, the dye becomes incorporated intracellularly into the endosomes and Golgi, and subsequently into the vacuoles (Fischer-Parton et al., 2000); it has been shown that endocytosis mutants show delayed or fragmented staining of intracellular vesicles (Gachet and Hyams, 2005; Martin et al., 2005). *N. crassa* wild type germlings readily showed plasma membrane and intracellular staining and round membranous structures were observed in the cytoplasm as early as at ~9.5 min after the addition of FM4-64 (Video 1 and Figure 3A). After 30 min of incubation with FM4-64, wild type cells displayed large FM4-64-positive membranous structures (Figure 3B). In contrast, the intracellular accumulation of FM4-64 in Δ*whi-2* cells was delayed and appeared as small elements, typical of fungal mutants with endocytosis defects (Gachet and Hyams, 2005; Martin et al., 2005) (Video 1 and Figures 3A,B).

*S. cerevisiae whi2* mutants show highly fragmented mitochondria or agglomeration of mitochondria in ball-like structures, depending on the growth phase (Leadsham et al., 2009; Mendl et al., 2011). We used a strain harboring a *gfp*-tagged *arg-4* (encoding the mitochondrial acetylornithine-glutamate acetyltransferase) (Bowman et al., 2009) to image the mitochondrial network in *N. crassa* germlings. In wild type germlings, mitochondria were abundant and formed a tubular-shaped network (Figure 3C). During cell communication and germling fusion, the shape of the mitochondrial network remained unchanged, although organelles were excluded from the tip of CATs until fusion and cytoplasmic continuity were attained (Figure 3D and Video 2). In contrast to the mitochondrial organization in wild type cells, Δ*whi-2* germlings displayed aberrant mitochondria that appeared to be fragmented and clustered into “masses” (Figure 3C). These observations indicated that WHI-2 was required for normal endocytosis and mitochondrial morphology in *N. crassa*.

### CSP-6 and AMPH-1 Are Two Fusion Proteins Whose Function Is Related to WHI-2

**Figure 4:**
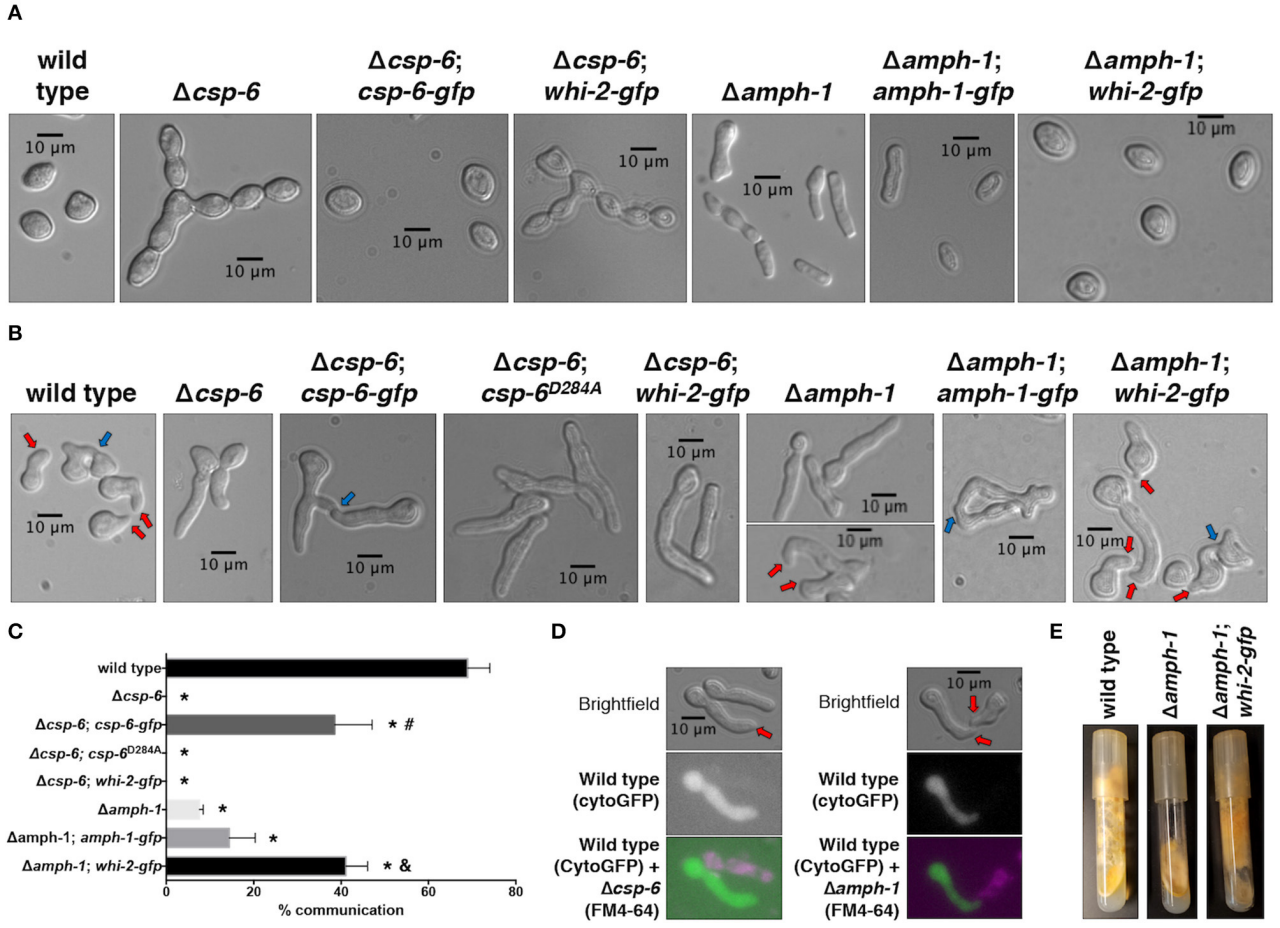
The mis-expression of *whi-2* in cells lacking *amph-1* but not *csp-6* compensates for germling communication and asexual defects. **(A)** Conidial morphology of indicated strains was analyzed by brightfield microscopy. **(B)** Cell-cell communication between cells from single strains (genetically identical) were evaluated between closely positioned germlings (**B**, 4 h post-inoculation). In **(B)**, red and blue arrows indicate conidial anastomosis tubes (CATs) and communication/fusion events, respectively. Note the presence of CATs in the Δ*amph-1* strain (lower micrograph, red arrows). The relative frequency of cell-cell communication of strains shown in **(B)** is shown in **(C)**. Error bars represent the standard deviation.*p-value < 0.0001 vs. wild type; #*p*-value < 0.0001 vs. Δ*csp-6*; and, *p*-value < 0.0001 vs. Δ*amph-1*. **(D)** Cytoplasmic GFP-expressing wild type cells were mixed with FM4-64-stained Δ*csp-6* or Δ*amph-1* cells and observed by microscopy after germination. Note the formation of CATs (red arrows). **(E)** The production of aerial hyphae and sporulation was evaluated after 7 days of growth for the indicated strains.

In a previous deletion strain screening, *csp-6* and *amph-1* were also found to be required for cell fusion in *N. crassa* (Fu et al., 2011, 2014; Chinnici et al., 2014). In addition, Δ*csp-6* and Δ*amph-1* cells also have a defect in conidial separation (Figure 4A and Figure S2), phenotypically similar to proconidial chains in Δ*whi-2* mutants (Figure 1C) (Fu et al., 2014; Ghosh et al., 2014). CSP-6 (NCU08380) is a Haloacid Dehalogenase (HAD) family Ser/Thr phosphatase and the ortholog of Psr1/2 from *S. cerevisiae*. In yeast and in *N. crassa*, WHI-2 and CSP-6 have been shown to directly interact (Kaida et al., 2002; Zhou et al., 2018). AMPH-1 (NCU01069) harbors an Arfaptin Homology/Bin-Amphiphysin-Rvs (AH/BAR) domain; AMPH-1 homologs in *S. cerevisiae* (Rvs161p and Rvs167p) regulate the actin cytoskeleton, endocytosis and mating, while in neurons AMPH-1 plays a role in clathrin-mediated endocytosis (Takei et al., 1999; Friesen et al., 2006). Although phenotypically similar, the growth of the Δ*amph-1* mutant was more affected than growth of the Δ*whi-2* or Δ*csp-6* mutants (Figure S2).

We confirmed that cell communication in populations of Δ*csp-6* germlings was absent, while a low frequency of CAT formation was observed in self-pairings of Δ*amph-1* germlings (Figures 4B,C). The introduction of a *gfp-*tagged *csp-6* or *amph-1* allele partially compensated for the developmental defects observed in Δ*csp-6* and Δ*amph-1* mutants, respectively (Figures 4A–C and Figure S2). Since the complementation of *amph-1* with the *gfp-*tagged *amph-1* allele was modest (Figure 4C), we conducted a co-segregation analysis by crossing the wild type and Δ*amph-1* deletion strains and analyzing the resistance of the progeny to hygromycin B; all strains that displayed wild type-like growth were sensitive to hygromycin B whereas all strains with flat-like growth were resistant to it (Figure S3). The Δ*csp-6* and Δ*amph-1* mutants communicated with wild type germlings, although at low frequency (Figure 4D); this observation had been previously suggested for Δ*amph-1* (Fu et al., 2014).

To explore how the predicted phosphatase activity of CSP-6 plays a role during cell fusion and asexual development in *N. crassa*, we constructed a mutated allele of CSP-6 predicted to abolish activity. In *S. cerevisiae*, a point mutation in the predicted catalytic aspartic acid residue of the hhh*D*xDx(T/V) motif—where “h” is a hydrophobic residue and “x” is any residue—abolished phosphatase activity (Rebay, 2015). This aspartic acid residue belongs to the HAD domain and is conserved in *N. crassa*. A Δ*csp-6* mutant that carried a phosphatase-dead, P*ccg-1*-driven *csp-6*^D284A^ allele showed the same defects as the Δ*csp-6* deletion strain, namely the lack of aerial hyphae (Figure S2) and inability to form CATs and undergo cell fusion (Figures 4B,C).

To assess the relationship between *whi-2, amph-1*, and *csp-6*, we placed an extra copy of *whi-2* under the control of a constitutive promoter (P*ccg-1*) in the Δ*amph-1* and Δ*csp-6* strains. The Δ*amph-1;*P*ccg-1-whi-2* strain showed suppression of the conidial separation defect of the Δ*amph-1* mutant (Figure 4A), restoration of CAT formation and chemotropic interactions (Figures 4B,C), and normal aerial hyphae development (Figure 4E). In contrast, over-expression of *whi-2* did not complement the morphological (Figure S2) or communication defects of the Δ*csp-6* mutant (Figures 4A–C). These data showed that the deletion of *whi-2, csp-6*, and *amph-1* leads to similar cellular phenotypes and that the mis-expression of *whi-2* compensated for the absence of *amph-1*, indicating that AMPH-1 functions upstream of WHI-2.

**Figure 5:**
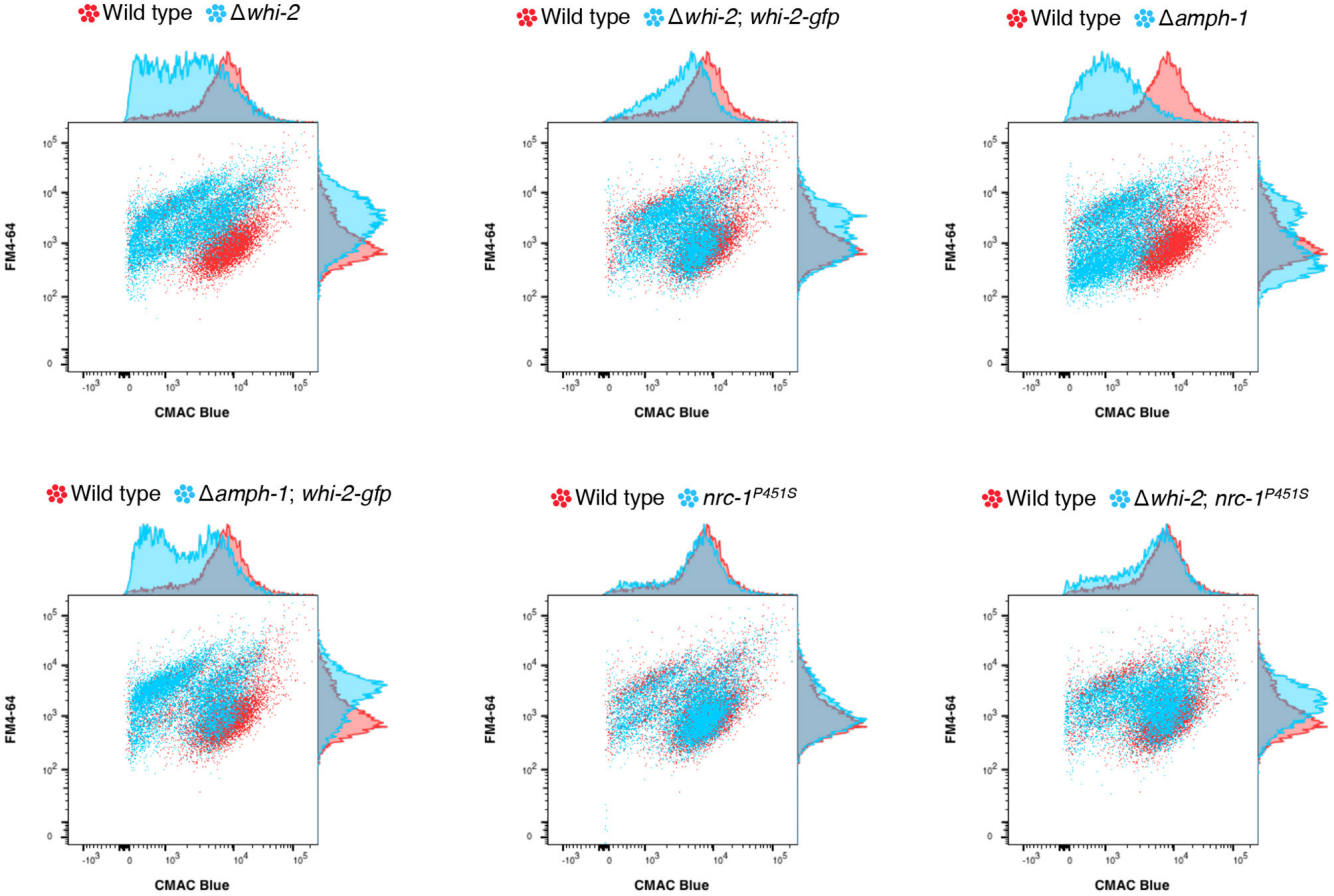
The mis-expression of *nrc-1*^*P*451*S*^ and *whi-2-gfp* compensates for the endocytosis defect of Δ*whi-2* and Δ*amph-1* cells, respectively. The wild type (red) and other indicated strains (blue) were stained with FM4-64 and CMAC Blue to label endocytic intermediates and vacuolar lumens, respectively. Note that while the wild type shows an overlap of FM4-64 and CMAC Blue, indicating that plasma membrane-derived endocytic intermediates are guided to the vacuoles, cells lacking *whi-2* and *amph-1* displayed a shift in red/blue fluorescence. n=4. Shown is a representative set of experiments.

In order to examine if the deletion of *amph-1* results in an endocytic defect, as predicted from *S. cerevisiae* literature (Munn et al., 1995; Kaksonen et al., 2005), and whether the mis-expression of *whi-2* also compensated for an endocytosis defect, we employed a flow cytometry-based methodology. The combined labeling of endocytic intermediates and vacuolar lumens (with FM4-64 and carboxydichlorofluorescein diacetate (CDCFDA), respectively) has been previously used to isolate *S. cerevisiae* mutants unable to properly execute endocytosis and transport plasma membrane material to the vacuole (Zheng et al., 1998). Such mutants display a shift in FM4-64/CDCFDA fluorescence as compared to wild type cells. We adopted a similar strategy, but utilized CMAC Blue to stain the lumen of the vacuoles instead of CDCFDA. The deletion of *whi-2* or *amph-1* resulted in a shift in FM4-64/CMAC Blue fluorescence profile as compared to wild type cells (Figure 5). However, the Δ*whi-2* and Δ*amph-1* strains carrying P*ccg-1*-*whi-2-gfp* showed a profile more similar to wild type cells (Figure 5). These data suggest that endocytosis defect was at least partially compensated for by the mis-expression of *whi-2-gfp* in Δ*amph-1* cells. Additionally, the FM4-64/CMAC Blue fluorescence shift in Δ*whi-2* cells was also partially recovered by the mis-expression of *nrc-1*^*P*451*S*^ (Figure 5), consistent with suppression of cell fusion defects in Δ*whi-2* cells (Figure 1).

### WHI-2 and CSP-6 Localize Mainly to the Cell Periphery While AMPH-1 Is Present in Cortical Patches and Puncta

**Figure 6:**
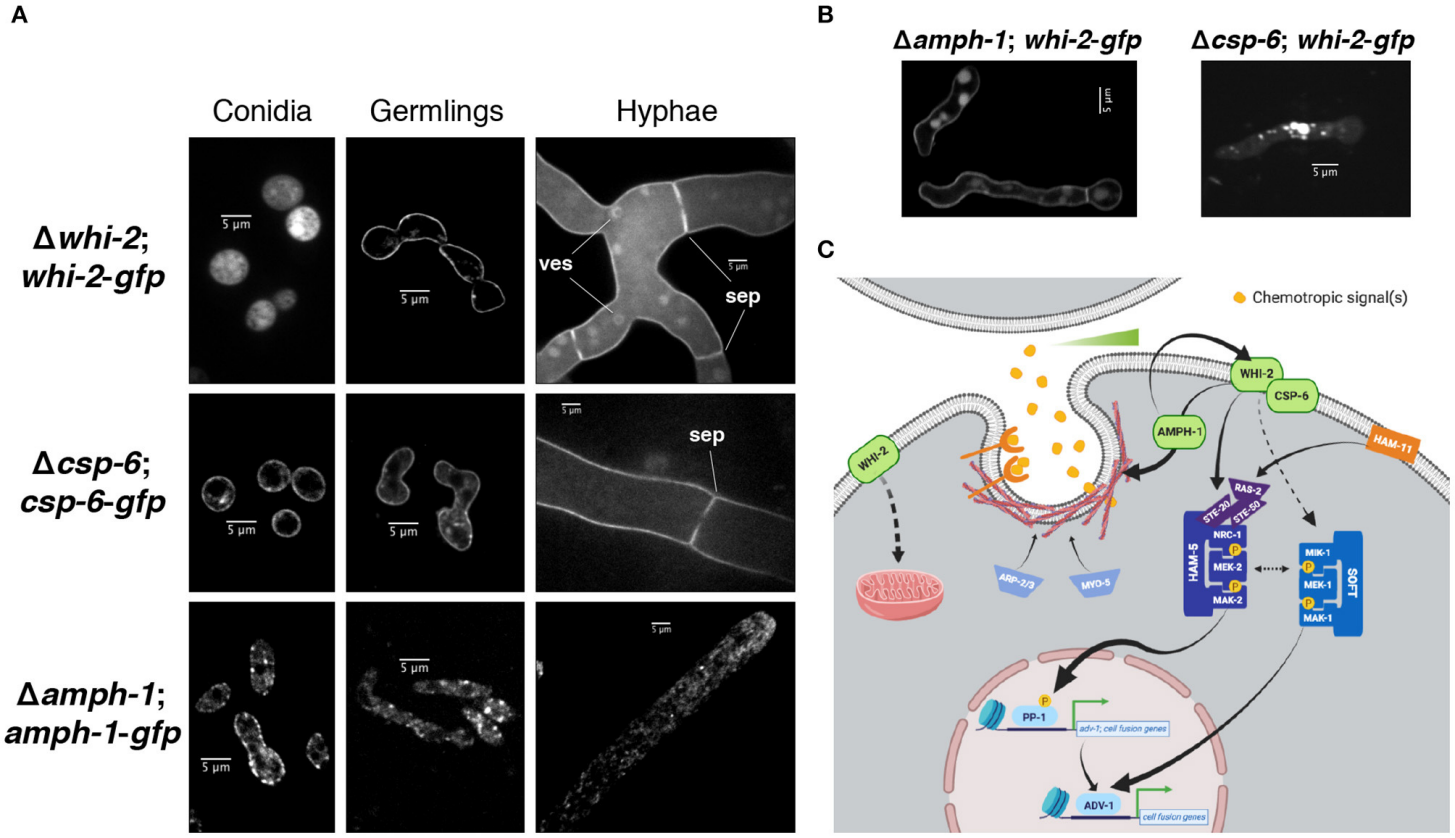
Localization of WHI-2, CSP-6 and AMPH-1 at different asexual stages. **(A)** The localization of WHI-2 (upper panel), CSP-6 (middle) and AMPH-1 (lower) was examined in the deletion strains carrying a GFP-tagged construct of the respective protein. Panels from left to right indicate conidia, germlings and mature hyphae. ves, intracellular vesicle membrane; sep, septum. **(B)** Localization of WHI-2 in Δ*amph-1* and Δ*csp-6* mutant background. **(C)** Schematic model of the role of WHI-2, CSP-6, and AMPH-1 in signal reception during cell-cell communication of *N. crassa* germlings. Sensing of a gradient of the chemotropic signal(s) may occur via endocytosis of the chemotropic signal or via internalization of a receptor. WHI-2 and CSP-6 interact and sit at the cell periphery. AMPH-1 could be involved in vesicle scission during endocytosis and is epistatic to WHI-2. WHI-2 may also play a role in one or more steps of endocytosis, potentially via AMPH-1. WHI-2 functions upstream of the NRC-1/MEK-2/MAK-2 signaling pathway. Previous western blot data (Fu et al., 2014) indicated that WHI-2 could also affect signaling through another MAPK pathway composed of a MIK-1/MEK-1/MAK-1 module. These two MAPK cascades are involved in the activation of the transcription factors PP-1 and ADV-1, which directly regulate the expression of genes involved in cell communication and fusion. WHI-2 also controls the organization of the intracellular mitochondrial network. For simplicity, a number of signaling components and phosphorylation events have been left out (Please see Fischer and Glass, 2019, for an extended review).

Previous reports showed that WHI-2 localized by immunofluorescence to the cytoplasm, small vesicles or vacuoles and by subcellular fractionation to the cytoplasmic and nuclear fractions; AMPH-1 immunolocalization studies showed a punctate pattern suggestive of small vesicles and by subcellular fractionation CSP-6 was present in the cytoplasmic and nuclear fractions (Fu et al., 2014; Zhou et al., 2018). However, the AMPH-1-RFP construct only marginally complemented the fusion defect of Δ*amph-1* (Fu et al., 2014), the immunofluorescence for WHI-2 was not very resolved (Fu et al., 2014) and the subcellular fractionation for WHI-2 and CSP-6 showed that both proteins were in all fractions tested (Zhou et al., 2018). Therefore, we sought to determine the localization of these proteins using confocal microscopy by tagging them with GFP, under the control of the constitutive P*ccg-1* promoter, and introducing them into their respective deletion strains (Figures 1, 4). In dormant conidia, WHI-2-GFP was distributed across the cytoplasm while CSP-6-GFP was predominantly present in the cell periphery (Figure 6A). AMPH-1 was mainly localized in cortical patches although some cytoplasmic staining was observed (Figure 6A). In germlings, WHI-2 and CSP-6 were present mainly in the cell periphery and occasionally in intracellular puncta (Figure 6A); AMPH-1 was present in cytoplasmic puncta. In mature hyphae, the localization of WHI-2 and CSP-6 was mainly at the cell periphery and at septa (Figure 6A). For WHI-2, we also observed cytoplasmic localization and in the membrane of round intracellular vesicles (Figure 6A). In hyphae, AMPH-1 was present in puncta or small intracellular vesicles that tended to accumulate at the hyphal tip (Figure 6C).

A Δ*amph-1*;*whi-2-gfp* strain was able to communicate and fuse (Figures 4B,C), but a Δ*csp-6*;*whi-2-gfp* strain displayed the same morphological defects as its parental deletion mutant (Figures 4B,C). We therefore assessed whether localization of WHI-2-GFP was affected in the Δ*amph-1* mutant relative to the Δ*csp-6* mutant and wild type cells. In the Δ*amph-1* mutant, WHI-2 displayed a cell periphery localization, consistent with what was observed in a Δ*whi-2*;*whi-2-gfp* strain (Figure 6A). However, in the Δ*csp-6* mutant, WHI-2-GFP was no longer present in the cell periphery, but accumulated in cytoplasmic patches and puncta (Figure 6B). These data suggest that CSP-6 is required for localization of WHI-2, consistent with reports of physical interaction of these two proteins in *N. crassa* and *S. cerevisiae* (Kaida et al., 2002; Zhou et al., 2018).

## Discussion

Germinated asexual spores of *N. crassa* cooperate during the formation of the somatic mycelial network by undergoing regulated cell fusion. Our study highlights the importance of WHI-2, an ortholog of the *S. cerevisiae* Whi2p, which binds to Psr1p (HAD family of protein phosphatases; CSP-6 ortholog) and regulates the response to nutritional stress by affecting both TORC1 and the Ras-cAMP-PKA pathway (Sudbery et al., 1980; Kaida et al., 2002; Müller and Reichert, 2011; Chen et al., 2018). The deletion of *whi-2* in *N. crassa* resulted in a panoply of morphological phenotypes, including defects in conidial separation, an inability to undergo chemotropic interactions and cell fusion, aerial hyphae formation, growth rate and female fertility. Our data showed that the lack of *whi-2* also caused an accumulation of FM4-64-positive membranous structures that likely correspond to endocytic intermediates and the destabilization of the mitochondrial organization. Using a constitutive *nrc-1* allele, we showed that WHI-2 plays an active role during cell-cell communication in *N. crassa* by transducing signals to the NRC-1/MEK-2/MAK-2 pathway. In support of this conclusion, the deletion of *whi-2, csp-6* or *amph-1* abolishes activation of *prm-1* (Fischer et al., 2019), which encodes a protein involved in plasma membrane merger during cell fusion (Fleissner et al., 2009a). The *prm-1* gene is directly regulated by the transcription factors PP-1 and ADV-1, which sit downstream of the NRC-1/MEK-2/MAK-2 pathway (Fischer et al., 2018). These data suggest that WHI-2 functions upstream in the sequence of signal transduction events that lead to chemotropism and cell fusion (Figure 6C). In yeast, Whi2p is a nutrient sensor (Müller and Reichert, 2011; Chen et al., 2018), and in mammalian models a specific form of endocytosis is regulated by nutrient availability (Pang et al., 2014). In *N. crassa*, the Δ*whi-2* mutant was phenotypically similar when grown under various carbon and nitrogen sources, indicating further biochemical and genetic analyses are required to assess whether WHI-2 affects nutrient sensing. It will be also interesting to examine the range of phenotypes controlled by the WHI-2/MAPK axis by taking the advantage of the Δ*whi-2*; *nrc-1*^*P*451*S*^ strain.

Although endocytosis is required for proper morphogenesis and apical growth in filamentous fungi (Riquelme et al., 2018), it has been suggested that these organisms might not undergo clathrin-dependent endocytosis (Schultzhaus et al., 2017). An alternative form of endocytosis termed fluid-phase endocytosis or pinocytosis, cells internalize small molecules present in the extracellular fluid through invaginations of the plasma membrane, forming intracellular vesicles (Epp et al., 2013) and a role for Whi2p during pinocytosis has been suggested as a way to sense cell density in yeast (Care et al., 2004). Additionally, this form of endocytosis has been proposed to mediate cell-cell communication between animal alveolar macrophages (Schneider et al., 2017). Pinocytosis could function as a mechanism to survey the extracellular space for gradients of certain peptides or metabolites and whenever the concentration is above a defined threshold, as when cells that produce a communication signal are in close proximity, the activation of the cell-cell fusion machinery would ensue.

Evidence from various organisms support the hypothesis that endocytosis could play a role in cell-cell communication in *N. crassa* (Figure 6C). In animals, fusogens are internalized via RAB-5 and DYNAMIN-1 GTPases-dependent clathrin-mediated endocytosis (Shin et al., 2014; Smurova and Podbilewicz, 2016). In the filamentous fungus *Ustilago maydis*, the pheromone receptor Pra1 cycles between the early endosomes and the plasma membrane in endocytic vesicles, triggering cell communication-associated MAPK signaling pathways when the mating pheromone is perceived. Mutations that impair endocytosis cause Pra1 to be depleted from the plasma membrane and abolish cell fusion (Fuchs et al., 2006). During mating in *S. cerevisiae*, the pheromone receptors and pheromone transporter are subject to regulation by endocytosis (Davis et al., 1993; Berkower et al., 1994).

Our observations that AMPH-1 is epistatic to WHI-2 adds strength to the hypothesis that endocytosis could be important for cell-cell communication in *N. crassa*. The yeast ortholog of AMPH-1, Rvs161p (previously termed Fus7p and End6p) is involved in endocytosis (Munn et al., 1995; Kaksonen et al., 2005) and forms a heterodimer with another BAR domain-containing protein, Rvs167p, to bind to phospholipid membranes (Friesen et al., 2006). BAR domains modulate membrane curvature by promoting vesicle scission at the neck of plasma membrane invaginations during endocytosis (Takei et al., 1999; McMahon and Gallop, 2005). Rvs161p also plays a role in yeast mating cell fusion by binding and shuttling Fus2p from the nucleus to the shmoo tip and by recruiting Cdc42p to the cell-cell contact spot (Smith et al., 2017); orthologs of Fus2p are absent in *N. crassa*. Despite the role of Rvs161p during mating cell fusion, some mutations in *RVS161* cause a defect in endocytosis but not in mating (Brizzio et al., 1998). Further investigations are required to ascertain if the situation in *N. crassa* is analogous. In addition to AMPH-1, mutations in other components of the endocytosis machinery in *N. crassa*, namely MYO-5 (Dettmann et al., 2014; Ramírez-Del Villar et al., 2019) and the ARP-2/ARP-3 complex proteins (Roca et al., 2010), also result in cell-cell communication defects (Figure 6C).

A strain containing a deletion of *csp-6* phenocopied a Δ*whi-2* mutant. WHI-2 physically interacts with CSP-6 to dephosphorylate the circadian clock regulator WC-1, which in turn results in loss of activation of ADV-1 (Zhou et al., 2018); ADV-1 directly regulates cell communication and fusion genes in *N. crassa* (Fischer et al., 2018). Our localization studies showed that WHI-2 and CSP-6 share the same subcellular distribution at the cell periphery. This observation supports our results that put WHI-2 and CSP-6 upstream of a signaling cascade that activates ADV-1. Importantly, a CSP-6 allele that lacks phosphatase activity was unable to complement the defects of a strain lacking *csp-6*, indicating that the phosphatase function of this protein is essential for CSP-6 function.

Although a receptor-mediated system where an extracellular receptor binds a chemotropic ligand has been suggested to underlie cell-cell communication in *N. crassa*, we speculate that endocytosis could be an important mechanism of signal sensing (Figure 6C). The peripheral localization of WHI-2, in line with previous results in *S. cerevisiae* (Huh et al., 2003), fits our proposed role in endocytosis, since this is where the endocytic machinery is recruited to initiate vesicle budding. WHI-2 and CSP-6 may be regulators of this machinery, act as sensors, or both; AMPH-1 is potentially involved in endocytosis vesicle scission (McMahon and Gallop, 2005). The fact that a Δ*whi-2* mutant is capable of a low level of communication with the wild type strain, including the recruitment of MAK-2 and SO to the fusion tips, suggests that the absence of *whi-2* does not hinder the production of pro-fusion cues, attracting the wild type partner, but may obstruct proper sensing. Signal receiving may be desensitized in the Δ*whi-2*, Δ*csp-6*, and Δ*amph-1* mutants and perhaps can only occur when the levels of chemotropic ligand(s) that are much higher than the levels required for communication in wild type cells. Only a single additional mutant previously identified in *N. crassa, ham-11*, shows a defect in self-fusion, but can undergo robust chemotropic interactions and fusion with wild type cells (Leeder et al., 2013; Fischer et al., 2019). Recent evidence suggests that HAM-11 also functions upstream of the NRC-1/MEK-2/MAK-2 signal transduction pathway (Fischer et al., 2019). HAM-11 is predicted to encode a hypothetical plasma membrane protein enriched in plasma membrane fractions. These data and data presented here suggest the AMPH-1, CSP-6-WHI-2, and HAM-11 function upstream of the MAK-2 signaling complex and play a role in sensitizing cells for signal reception during chemotropic interactions.

## Data Availability Statement

All datasets generated for this study are included in the article/Supplementary Material.

## Author Contributions

AG and NG designed the study and wrote the article. AG performed all experiments. KC contributed to the quantification of cell communication. SC-S contributed to the flow cytometry assay and mutant segregation analysis.

### Conflict of Interest

The authors declare that the research was conducted in the absence of any commercial or financial relationships that could be construed as a potential conflict of interest.
