# Supplementary material for: WHI-2 Regulates Intercellular Communication via a MAP Kinase Signaling Complex
Source: Front Microbiol. 2020 Jan 22;10:3162. doi: 10.3389/fmicb.2019.03162 (PMC6987382; doi:10.3389/fmicb.2019.03162)
Supplement: Supplementary file 3 [file Data_Sheet_1.pdf]

Supplementary Material

Table S1 – *Neurospora crassa* strains used in this study.

Name	Genotype	Source
Wild type (74-OR23-IV) – FGSC2489	*mat A*	FGSC
Wild type (ORS-SL6a) – FGSC4200	*mat a*	FGSC
his-3 – FGSC6103	*his-3*; *mat A*	FGSC
his-3 – FGSC9716	*his-3*; *mat a*	FGSC
Wild type; CytoGFP	*his-3*::P*ccg-1*-*gfp*-T*ccg-1*	(Fleissner et al., 2009a)
Δ*whi-2* A	ΔNCU10518	This study
Δ*whi-2* a	ΔNCU10518	This study
Δ*whi-2*; *his-3*	ΔNCU10518; *his-3*	This study
WHI-2-GFP	ΔNCU10518; *his-3*::P*ccg-1*-*NCU10518*-*gfp*-T*ccg-1*	This study
Δ*csp-6* A	ΔNCU08380	This study
Δ*csp-6* a	ΔNCU08380	This study
Δ*csp-6*; *his-3*	ΔNCU08380; *his-3*	This study
CSP-6-GFP	ΔNCU08380; *his-3*::P*ccg-1*-*NCU08380*-*gfp*-T*ccg-1*	This study
CSP-6^{D284A}-GFP	ΔNCU08380; *his-3*::P*ccg-1*-*NCU08380(695A>C)*-*gfp*-T*ccg-1*	This study
Δ*amph-1* A	ΔNCU01069	This study
Δ*amph-1* a	ΔNCU01069	This study
Δ*amph-1*; *his-3*	ΔNCU01069; *his-3*	This study
AMPH-1-GFP	ΔNCU01069; *his-3*::P*ccg-1*-*NCU01069*-*gfp*-T*ccg-1*	This study
Δ*csp-6*; WHI-2-GFP	ΔNCU08380; *his-3*::P*ccg-1*-*NCU10518*-*gfp*-T*ccg-1*	This study
Δ*amph-1*; WHI-2-GFP	ΔNCU01069; *his-3*::P*ccg-1*-*NCU10518*-*gfp*-T*ccg-1*	This study
NRC-1^{P451S}	*his-3*::P*ccg-1*-*3xflag*-*NCU06182(1351C>T)*	(Dettmann et al., 2012)
Δ*whi-2*; NRC-1^{P451S}	ΔNCU10518; *his-3*::P*ccg-1*-*3xflag*-*NCU06182(1351C>T)*	This study
Wild type; SO-dsRed	*his-3*::P*ccg-1*-*dsRed*-*NCU02794*-T*ccg-1*	(Fleissner et al., 2009b)
Wild type; MAK-2-mCherry	*his-3*::P*ccg-1*-*NCU02393*-*gfp*-T*ccg-1*	(Fleissner et al., 2009b)
Δ*whi-2*; SO-GFP	ΔNCU10518; *his-3*::P*ccg-1*-*NCU02794*-*gfp*	This study
Δ*whi-2*; MAK-2-GFP	ΔNCU10518; *his-3*::P*ccg-1*-*NCU02393*-*gfp*	This study
Wild type; ARG-4-GFP	*his-3*::P*ccg-1*-*NCU10468*-*gfp*-T*ccg-1*	(Bowman et al., 2009)

FGSC: Fungal Genetics Stock Center.

```
NcWHI-2    1 -------MAAAGG--ASSIITQVQQGGPPINALGDVAPDETITMDLRGTRFTLSRDELLT
ScWhi2p    1 MDDIITQVSPDNAESAPILQEQQQQQNSQYEGNEEDYGDSLIHLNIQENHYFITRDQLMS

NcWHI-2   52 LPEFVLLSLFPNGLFPEGHMGGF----GEGDAVQVDYDPASLQYMLEFFRTVAQSIPVDP
ScWhi2p   61 LPESLLLCLFPSGVFLDRCGQVITNLTRDDEVYIVNFPPDCFEYIMEIYTKAHDDLYNHP

NcWHI-2  108 SNPQ---------DGSDGIVPVD---P-ASGARGDDGSKRAGIIVLREDLDFYAIPPRAD
ScWhi2p  121 VEKFFDRPSSSFVSNAKGFFGLSSNNSISSNNEQDILHQKPAIIVLREDLDYYCVPQEEF

NcWHI-2  155 L------------GQAEMMEIKRAAARALLKQDGIFSGLK--------------------
ScWhi2p  181 QFDSTNEENNEDLLRHFMAQVKMAAGSYLTSKTSIFQGLYSSNRLKQQQQQKIEKGSNS
                                                           *
NcWHI-2  183 ------KSDEPGTTEAHLIEMLTAGGFNHDDRWGHRAGEPNKAVICSLALARLRSDIKGN
ScWhi2p  241 SSNTKSTSKKLGPAEQHLMDMLCSSGFTKETCWGNRTQETGKTVISSLSLCRLANETTEG

NcWHI-2  237 E---------------------------------------------------MG------
ScWhi2p  301 FRQKFNEAKAKWEAEHKPSQDNFITPMQSNISINSLSASKSNSTISTARNLTSGSTAPAT

NcWHI-2  240 -----------------------------------------SNAVGMAQKLLLFWRKPAR
ScWhi2p  361 ARDKRKSRLSKLADNVRSHSSSRHSSQTRSKPPELPKLYDLVPKPNINAKLLLFWRKPAR

NcWHI-2  259 RCWWEGVELEG-VE-------------------GLEP-------GTKLKVWIRRVWTLEM
ScWhi2p  421 KCWWGEEDIELEVEVFGSWKDESKKIIELILPTNVDPEAELHKIIVPVRLHIRRVWTLEL

NcWHI-2  292 SVIGLR 297     Percent identity: 24.02%
ScWhi2p  481 SVIGVQ 486   Percent similarity: 33.47%
```

Figure S1. WHI-2 is the ortholog of S. cerevisiae Whi2p.
Alignment of the amino acid sequences of YOR043W (Whi2p, *S. cerevisiae*) and NCU10518 (WHI-2, *N. crassa*). The two BTB/POZ domains of WHI-2 are delimited by a rectangle.

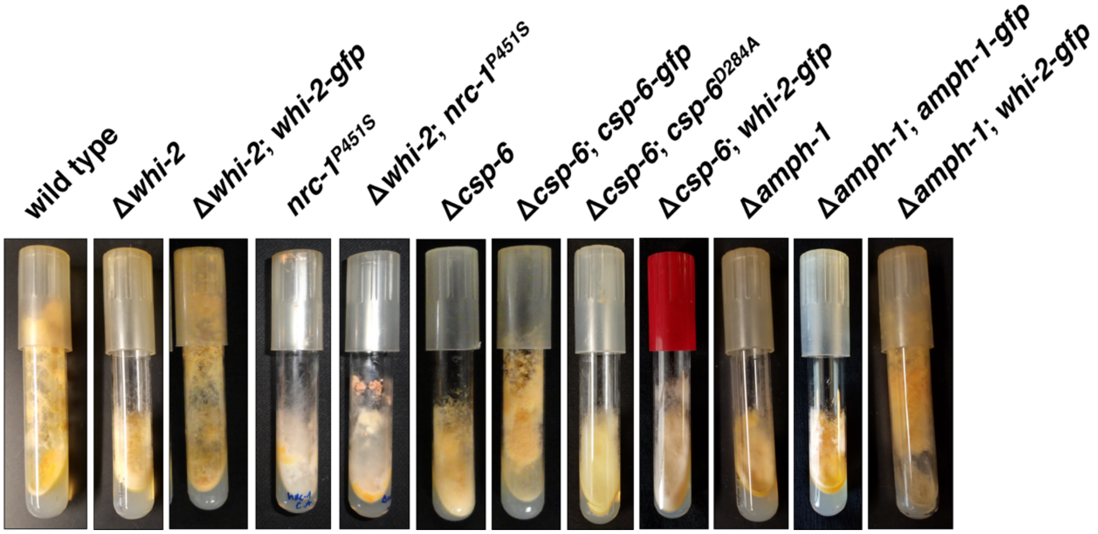

Figure S2. Aerial hyphae phenotype of strains used during the course of this work. The production of aerial hyphae and sporulation was evaluated after 7 days of growth for the indicated strains.

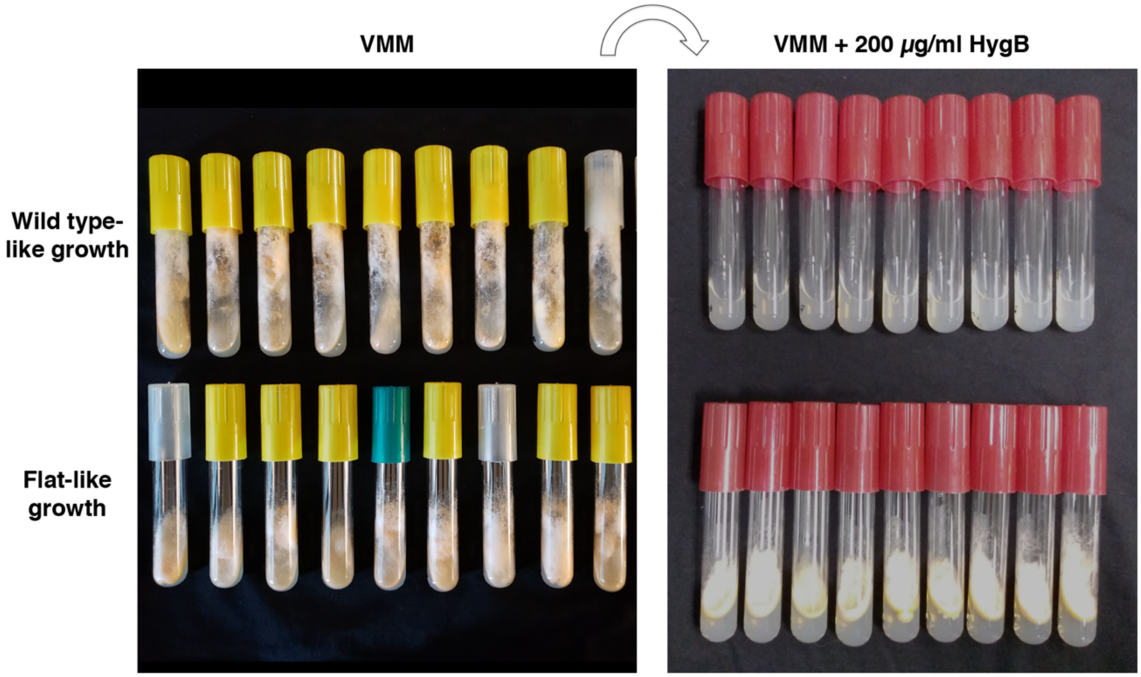

Figure S3. Co-segregation analysis of a wild type x Δ*amph-1* cross.
The wild type strain (FGSC2489) and the Δ*amph-1* mutant strain were crossed and a set of 18 progeny strains were grown on VMM and subsequently passaged to VMM containing hygromycin B (HygB). Note that only strains that displayed a flat-like growth on VMM were capable of growing when transferred to hygromycin B medium.

Movie 1. Endocytosis kinetics in wild type *versus* Δ*whi-2*. A white arrow indicates the moment when large round FM4-64-labeled vesicles are observed in the wild type strain.

Movie 2. Mitochondrial dynamics during fusion of wild type cells. Note the absence of ARG-4 localization to the zone of cell-cell interaction until fusion has been attained.

References

Bowman, B.J., Draskovic, M., Freitag, M., and Bowman, E.J. (2009). Structure and distribution of organelles and cellular location of calcium transporters in *Neurospora crassa*. *Eukaryot Cell* 8(12)**,** 1845-1855. doi: 10.1128/EC.00174-09.

Dettmann, A., Illgen, J., Marz, S., Schurg, T., Fleissner, A., and Seiler, S. (2012). The NDR kinase scaffold HYM1/MO25 is essential for MAK2 map kinase signaling in *Neurospora crassa*. *PLoS Genet* 8(9)**,** e1002950. doi: 10.1371/journal.pgen.1002950.

Fleissner, A., Diamond, S., and Glass, N.L. (2009a). The Saccharomyces cerevisiae PRM1 homolog in *Neurospora crassa* is involved in vegetative and sexual cell fusion events but also has postfertilization functions. *Genetics* 181(2)**,** 497-510. doi: 10.1534/genetics.108.096149.

Fleissner, A., Leeder, A.C., Roca, M.G., Read, N.D., and Glass, N.L. (2009b). Oscillatory recruitment of signaling proteins to cell tips promotes coordinated behavior during cell fusion. *Proc Natl Acad Sci U S A* 106(46)**,** 19387-19392. doi: 10.1073/pnas.0907039106.
